# Mental Number Representations in 2D Space

**DOI:** 10.3389/fpsyg.2019.00172

**Published:** 2019-02-05

**Authors:** Elena Sixtus, Jan Lonnemann, Martin H. Fischer, Karsten Werner

**Affiliations:** ^1^Faculty of Human Sciences: Research Group “Motor Control and Cognition,” University of Potsdam, Potsdam, Germany; ^2^Empirical Childhood Research, University of Potsdam, Potsdam, Germany; ^3^Division of Cognitive Sciences, University of Potsdam, Potsdam, Germany

**Keywords:** spatial-numerical associations, SNARC, vertical space, horizontal space, Go/No-go task

## Abstract

There is evidence both for mental number representations along a horizontal mental number line with larger numbers to the right of smaller numbers (for Western cultures) and a physically grounded, vertical representation where “more is up.” Few studies have compared effects in the horizontal and vertical dimension and none so far have combined both dimensions within a single paradigm where numerical magnitude was task-irrelevant and none of the dimensions was primed by a response dimension. We now investigated number representations over both dimensions, building on findings that mental representations of numbers and space co-activate each other. In a Go/No-go experiment, participants were auditorily primed with a relatively small or large number and then visually presented with quasi-randomly distributed distractor symbols and one Arabic target number (in Go trials only). Participants pressed a central button whenever they detected the target number and elsewise refrained from responding. Responses were not more efficient when small numbers were presented to the left and large numbers to the right. However, results indicated that large numbers were associated with upper space more strongly than small numbers. This suggests that in two-dimensional space when no response dimension is given, numbers are conceptually associated with vertical, but not horizontal space.

## Introduction

The mental representation of numbers is a current and partly controversial subject. Especially studies regarding the SNARC (spatial-numerical association of response codes) effect are accumulating. Typically, such studies require participants to classify single digits presented at the center of a computer screen with speeded button responses as either odd or even. It is usually reported that in Western cultures, responses to numbers representing small magnitudes are faster on the left than right side and responses to numbers representing large magnitudes are faster on the right than left side (e.g., Dehaene et al., 1993; Schwarz and Keus, 2004; Keus and Schwarz, 2005; Wood et al., 2008; Hesse and Bremmer, 2017; Sixtus et al., 2017; Gökaydin et al., 2018; Lohmann et al., 2018). Typically, the SNARC effect is explained as reflecting a horizontal mental number line (MNL) with larger numbers to the right of smaller numbers for Western cultures. Previous research suggests that this horizontal association partly depends on individual experiences. One example of such experiences are cultural conventions such as reading direction (Dehaene et al., 1993; Shaki and Fischer, 2008; Shaki et al., 2009; Fischer et al., 2010; see Nuerk et al., 2015 for a discussion of mechanisms contributing to the influence of reading direction). Another relevant experience is finger counting, as indicated by an influence of the starting hand in finger counting on spatial-numerical associations (SNAs; Fischer, 2008; Fabbri, 2013; but see Sixtus et al., 2018). Moreover, there is evidence that there might be innate associations between magnitudes and horizontal space, both in humans and in animals (Rugani et al., 2015; de Hevia et al., 2017). In addition to this horizontal association of numbers and space, some studies investigated the diagonal, vertical, and radial relationship where usually numerically small numbers are associated with lower, lower left, and near space and numerically larger numbers with upper, upper right, and far space, respectively (e.g., Schwarz and Keus, 2004; Holmes and Lourenco, 2012; Sell and Kaschak, 2012; Fabbri, 2013; Grade et al., 2013; Winter and Matlock, 2013; Göbel, 2015; Winter et al., 2015; Hesse and Bremmer, 2017). Winter et al. (2015) discussed SNAs along three dimensions (horizontal, vertical, and radial) with an eye on potential origins of these different mappings and how these number mappings fit in with our current knowledge of brain organization and brain-culture interactions. Importantly, Fischer (2012; see also Fischer and Brugger, 2011; Pezzulo et al., 2011) suggested that the vertical association should be more stable than the horizontal one because it results from experience with laws of physics (e.g., a pile containing a larger number of objects is higher). Moreover, this vertical spatial association of magnitude is considered to be universal because physical laws apply regardless of cultural habits.

The present study aims to investigate conceptual spatial associations of numbers in two-dimensional space and in particular to compare relative association strengths along a horizontal and a vertical axis. “Conceptual” refers to the idea that spatial associations are an essential part of the meaning of numbers, rather than an extraneous and epiphenomenal part of number processing. In almost all studies, the way of assessing SNAs has not allowed for conclusions regarding conceptual SNAs because either the specific numerical magnitude or position in space (or both) have been explicit, task-relevant parts of experiments. Especially the association of numbers with horizontal space (i.e., spatial positions along a one-dimensional horizontal axis) has been investigated in a broad variety of tasks. In many paradigms, numbers are judged as regarding their parity (as odd or even) or magnitude (as smaller or larger than a reference number) and responses are given via buttons at the left and right side (ever since Dehaene et al., 1993). A magnitude judgment task with spatially distributed response buttons emphasizes both the magnitude represented by numbers and the spatial dimension along which numbers might be arranged. Thus, it addresses explicit magnitude processing and explicit spatial processing, which primes the spatial dimension along which response buttons are arranged within the task. In a study by Ranzini et al. (2016), participants were primed with a left- or rightward moving dot which they pursued with eye-movements and responded verbally (i.e., non-spatially) in a parity judgment task. Here, no spatial dimension was primed by responses and implicit magnitude processing was tested because parity judgments do not require magnitude information *per se*. However, the horizontal dimension was again clearly primed by the horizontally moving dot. There are many more examples of studies which reported typical horizontal and/or vertical SNAs in very different paradigms but primed the spatial dimension, for example by head turns (horizontal: Loetscher et al., 2008; Sosson et al., 2018; horizontal and vertical: Winter and Matlock, 2013), left- or right turns when walking (Shaki and Fischer, 2014), and saccades (horizontal: Fischer et al., 2004; horizontal and vertical: Schwarz and Keus, 2004).

A recent study by Shaki and Fischer (2018) shed some light on conceptual spatial associations of numbers by testing both magnitude processing and spatial processing implicitly. More precisely, the authors compared explicit and implicit magnitude processing by employing both a magnitude comparison and a parity judgment task, respectively. They furthermore used an implicit association task (IAT), that is, a Go/No-go task with only one central response button so that a number-space association could not be primed by the response dimension but was determined by response rules alone. Target stimuli were single-digit numbers and arrows pointing left/right/down/up with the horizontal and vertical dimension in separate blocks. Response rules always combined number magnitude or parity with a directional cue (e.g., respond only to odd numbers and to arrows pointing leftward). The idea was that implicit associations between numbers and spatial concepts (arrows pointing toward different directions) influenced response efficiency. Indeed, the authors found that reliable horizontal associations failed to appear for implicit magnitude processing while vertical associations persisted (in their Experiment 1). This suggests that numbers are conceptually associated with vertical space only and that horizontal associations are merely an artifact of the task ingredients of usual SNARC experiments (e.g., priming horizontal space with spatially distributed response buttons). The present study follows up on Shaki and Fischer’s (2018) approach of addressing both implicit magnitude processing and implicit spatial processing.

However, even in Shaki and Fischer’s (2018) approach, one may argue that either the horizontal or vertical dimension were primed because each response rule included only one spatial dimension among the target items. The present study combines the horizontal and vertical dimension within one paradigm and compares relative association strengths along the two axes when both dimensions are relevant at the same time. Additionally to Shaki and Fischer’s (2018) study, so far only few studies have compared horizontal and vertical associations of numbers and those employed both dimensions within separate blocks or combined both in the form of diagonal axes. Such studies moreover led to differing interpretations. We will shortly describe four relevant studies in the following paragraphs.

Winter and Matlock (2013) employed a random number generation task where participants were instructed to randomly generate (and state) numbers while alternately turning the head to the left and right – or down and up in a separate block. The authors analyzed the average difference between a generated number and the one generated during the preceding head turn. Analogously to typical horizontal and vertical SNARC effects with faster left than right responses as well as faster down than up responses to small numbers (and vice versa for large numbers), the authors expected smaller generated numbers after head turns toward to left side and downward, and larger generated numbers after head turns toward the right side and upward. Consistent with this hypothesis numbers generated during right- and upward head turns were on average significantly larger than the previously generated numbers. This effect was stronger in the vertical head turn condition than in the horizontal head turn condition, which suggests that vertical associations of numbers and space are stronger than horizontal associations. Blini et al. (2018) used optokinetic stimulation to induce shifts in spatial attention. Participants solved addition and subtraction problems during horizontal or vertical optokinetic stimulation. Besides specific effects of vertical optokinetic stimulation on decade errors during subtraction, gaze positions were influenced by operation type. Importantly, vertical eye movements were affected by operation type more reliably than horizontal eye movements, again suggesting that number processing (in this case addition and subtraction) interacts more strongly with vertical than horizontal spatial associations.

However, Holmes and Lourenco (2012) report contradictory results. The authors compared horizontal and vertical manual responses to numbers [0–9] in a parity judgment task as well as the two diagonal alignments of response positions. In their experiments, an Arabic digit appeared centrally on a touchscreen and responses were given via touches to visually presented response boxes below/above, left/right, left-below/right-above, or left-above/right-below the presented number. Importantly, the various response axes only appeared in separate blocks or experiments. A horizontal SNARC effect emerged with faster left than right responses to small numbers and faster right than left responses to large numbers, but no consistent vertical SNARC effect became evident. The diagonal SNARC depended on the horizontal axis, that is, SNAs apparently ran from left (down/up) for small numbers to right (up/down) for large numbers. Only in a second experiment when participants were instructed to imagine the numbers as floors in a building or levels of depth in a swimming pool did a vertical SNARC emerge. Hesse and Bremmer (2017) furthermore compared horizontal, vertical, and diagonal saccadic responses to numbers [1–9, except 5]. The parity status of numbers, which were presented visually and auditorily in separate blocks, had to be indicated via a saccade toward one of two dots that were positioned along a horizontal/vertical/diagonal axis. Again, only one axis was employed during an experimental block. The authors reported reliable SNARC effects with visually presented target numbers for the horizontal axis and the left-down to right-up diagonal axis. For the vertical axis, it was only present in error rates but not RTs and for the left-up to right-down diagonal axis it was not present at all.

Taken together, in the various studies, different pictures emerged depending on the task: it is unclear whether the vertical or horizontal representation of numbers is more reliable and, above all, it remains unclear whether the measured SNAs only arise as a result of the spatial presets of the concurrent task or response condition. Some indications of automatic co-activations among space and numerical magnitude are given by eye movement studies. Loetscher et al. (2010) found that in a random number generation task changes in horizontal and vertical eye position correlated with number magnitude: right- and upward eye movements predicted the generation of a number larger than the previous one and left- and downward eye movements predicted the generation of a number smaller than the previous one. Hartmann et al. (2016) found that counting upward induced shifts of eye position up and to the right, while results were unclear for the task of counting downward. In a study by Holmes et al. (2016), participants were (digitally) dealt cards in a blackjack game which required the mental addition of the cards’ numerical values. The authors found that the total numerical value of the dealt cards was reflected in the participants’ eye movements along the horizontal axis. These studies suggest a conceptual spatial association of numerical operations (random number generation, counting, addition). However, it remains unclear whether specific numbers are also linked to spatial positions.

Another open question concerns the generalizability of specific SNAs. Galton (1880; see also Seron et al., 1992) presents descriptions of spatial arrangements of mental number representations which had been reported to him by various people. Many of these arrangements differ a lot from each other and also from the arrangement along a straight horizontal or vertical number line which is usually assumed in more recent studies. In number-form synesthetes, these spatial associations of numbers have even been shown to affect psychometric measures in spatial-numerical tasks (Jarick et al., 2009). This illustrates that mental representations may vary substantially inter-individually and that individual mental representations can have objective effects in measures of mental number processing. A study by Fischer and Campens (2009) also shows different spontaneous orientations of the number line when blindfolded participants were instructed to indicate spatial positions of different numbers by pointing somewhere in the space in front of them. Even in these non-synesthetic participants various orientations of the number lines emerged (horizontal, vertical, and radial). Note also that it is usual in SNARC experiments that the typical SNARC effect appears in only a part of the participants (typically between 60–80%; cf. Cipora and Wood, 2017; Wood et al., 2006a,b, 2008) Furthermore, there might be concrete factors which influence the specific orientation of individual MNLs (for a recent review, see Toomarian and Hubbard, 2018). This has been reported for reading direction (Shaki and Fischer, 2008; Shaki et al., 2009; Fischer et al., 2010) and the starting hand in finger counting (Fischer, 2008; Fabbri, 2013). Regarding reading direction, Shaki et al. (2009) found a reverse SNARC effect in Palestinians who read words and numbers from right to left. Regarding finger counting habits, Fischer (2008) found that participants who started to count on the left hand (“left-starters”) but not those who started to count on the right hand (“right-starters”) showed a reliable horizontal SNARC effect in a parity judgment task. On the other hand, in Fabbri’s (2013) sample, right-starters showed a significantly stronger horizontal SNARC effect in a magnitude comparison task (but not in a parity judgment task). Although the results of these latter two studies do not fully converge, both suggest that experience with numbers through finger counting influences specific SNAs.

The main goal of the present study was to investigate the mental association between numbers and space in a two-dimensional grid of items when neither dimension (horizontal/vertical/diagonal) was primed by the task and when number magnitude was task-irrelevant. Avoiding a spatial distribution of response locations required a different method of integrating space into the task. In the present experiment, participants were therefore required to detect Arabic numbers within a grid of spatially distributed visual items and to respond with a single central response button. That way spatial congruency arose from the relationship between the numerical magnitude of the target number and the spatial position of visual target number presentation. Target numbers were auditorily primed before visual presentation and we expected that this auditory perception of numbers would co-activate associated spatial representations and would thereby influence search behavior and/or spatial attention. We included auditory primes that were numerically identical to the visual targets, so that number size could be expected to influence task performance before target detection. Thus, response efficiency at different spatial locations should be affected. Based on Holmes and Lourenco’s (2012) and Hesse and Bremmer’s (2017) findings, we would expect horizontal associations to be stronger than vertical associations. However, in these studies, spatially distributed responses were employed so that it is conceivable that the response dimension served as a prime for the number-space association (cf. Shaki and Fischer, 2018). Based on Shaki and Fischer’s (2018) results, on the other hand, we would expect only a vertical, but no horizontal association. The present experiment therefore tests their argumentation that without explicit magnitude processing and/or horizontally arranged responses numbers should only be associated with vertical but not with horizontal space. We furthermore inquired about participants’ finger counting habits to gain further insights into the relationship between individual finger-to-number mappings and SNAs.

## Materials and Methods

### Participants

Thirty-seven participants were tested in return for payment. Data from two participants were excluded because of technical issues (causing partial data loss) or high error rate (see section “Analysis”). Five were non-German native speakers and were also excluded from analyses because the paradigm included German prime words. Of the remaining 30 participants, 23 were female and the mean age was 25 years, *SD* = 6.95. Handedness was assessed by self-report: one was left-handed, the rest were right-handers. The study was reviewed and approved by the Ethics Committee of the University of Potsdam, all subjects gave written informed consent, and the experiment was conducted in accordance with the ethical standards expressed in the Declaration of Helsinki.

### Apparatus

Participants were individually tested while seated at a table facing a PC monitor. Visual stimuli were presented on the PC monitor (60 Hz refresh rate, 68.5 cm screen diagonal). Auditory stimuli were presented via headphones (AKG K-182; Harman Deutschland GmbH; Garching, Germany). The experiment was controlled and data recorded by expyriment software (Krause and Lindemann, 2014) on a laptop (Lenovo T430s, Stuttgart, Germany). Participants were seated so that they had a viewing distance of 60 cm from the monitor. Midsagittally in front of the participants was a custom-made wooden box containing a central single response button (28 mm diameter).

### Stimuli

Primes were auditorily presented German number words (1: “eins,” 2: “zwei,” 8: “acht,” 9: “neun”) with a duration of 500 ms each, spoken by a female voice. Target numbers were visually presented Arabic numerals within a grid of distractor symbols (“#”; text size of target and distractor symbols = 28 pixels, sans serif font type). Each target screen included 49 black symbols on a white background which were arranged as follows. Centrally on the screen was a square of 1100 × 1100 pixels which was again divided into 7 × 7 equal squares. Each of these 49 mini-squares contained one symbol (distractor or target number). The exact position of the symbol within the square was randomly selected with the only constraint that it had a distance of at least 10 pixels from the mini-square’s border to prevent overlap of the symbols. The borders of the mini-squares were not visible at any time.

### Procedure

**FIGURE 1:**
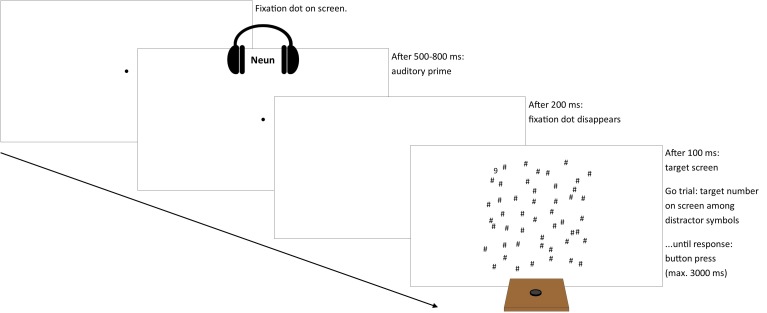
Illustration of trial sequence (stimuli not to scale).

Participants were instructed to place both hands centrally in front of them with the dominant hand on the response button. Each trial started with the presentation of a central fixation dot. After a random interval between 500 and 800 ms, the auditory prime number was presented and 200 ms thereafter the fixation dot disappeared. After another 100 ms, the target screen appeared (see Figure 1). Participants responded by button press whenever they detected the Arabic numeral among the distractors (Go trials). Reaction times (RTs) were defined as the duration between the target screen presentation and button press. In No-go trials, there was no Arabic numeral on the screen and participants should refrain from responding. Each trial ended with a button press or after 3000 ms. Visual feedback was given after erroneous responses. Whenever participants made errors in two consecutive trials, a warning screen advised concentration. In the very beginning, there was a short training.

After the main experiment, the experimenter inquired the participant’s spontaneous finger counting habits. She faced the participant, asked “Show me how you count from one to ten on your fingers,” and noted the fingers used, the order of fingers, and the starting hand.

### Design

Each target number (1, 2, 8, 9) appeared at each of the 49 positions three times. Thus, the experiment comprised 588 Go trials (49 positions × 4 target numbers × 3 repetitions). Within each sequence of five Go trials, a No-go trial was inserted at a random position. On average, every 6th No-go trial was followed by an additional No-go trial to avoid predictability of Go trials following No-go trials. Auditory stimuli for the No-go trials were randomly selected among the target numbers.

The training comprised 10 trials with at least four Go and four No-go trials. It could be repeated when necessary. The whole experiment took about 45 min.

### Analyses

Raw data and the analysis script are available online via https://osf.io/4agk5. Data of participants was excluded when the individual error rate in No-go trials exceeded the mean error rate in No-go trials among all participants plus/minus three standard deviations (SDs) because it indicates a tendency towards precocious responses without a genuine search of the target screen. This was the case for one participant with 25% erroneous No-go trials (mean error rate in No-go trials: 2.97%, *SD* = 4.92% for *N* = 37). After the exclusion of this participant, one participant with whom technical issues occurred, and the non-German native speakers (see section “Participants”) the mean error rate in Go trials was 1.58%, *SD* = 1.63% and in No-go trials 2.38%, *SD* = 3.53% (*n* = 30). Trials with RTs below 300 ms were excluded (0.02% of the data). Only Go trials were further analyzed. Inverse efficiency scores (IES; e.g., Townsend, 1983; Bruyer and Brysbaert, 2013) are reported in the first place instead of raw RTs, because IES better reflect performance while RTs of correct responses neglect the worse performance of incorrect (i.e., missed) responses. IES was calculated as mean RTs of correct responses divided by the percentage of correct responses (PC) per participant for each condition relevant in the respective analysis (e.g., mean RTs/PC for left presentations and for right presentations). They are reported with ms as units and can be interpreted similar to RTs, that is, the smaller the IES, the more efficient (faster and accurate) was the response. An important precondition for using IES is the absence of a speed-accuracy trade-off. This was confirmed by preliminary analyses: RTs and error rates per target number and presentation position (i.e., specific horizontal and vertical position within the 7 × 7 grid) were strongly positively correlated, ρ = 0.74, *t*(194) = 15.11, *p* < 0.001.

Being interested in SNAs similar to those reported in the literature on the SNARC effect, we aimed to follow the usual SNARC analyses. However, in contrast to usual SNARC experiments, the present experiment involved only one possible response with a central response button. Congruency arose from the relationship of numerical magnitude of the target number with the spatial position of visual target number presentation instead of the relationship of numerical magnitude of the target number with the spatial position of the response buttons. We analyzed both horizontal and vertical SNAs. The segmentation of the target screen into a 7 × 7 grid allowed for a more or less central presentation of targets along the horizontal and vertical axis (i.e., targets within the seven, either horizontally or vertically aligned middle mini-squares). Analyses of responses to left-/right-/down-/upward targets therefore excluded trials with targets on the respective central positions. Potential effects of SNAs were analyzed separately for horizontal and vertical spatial segmentation (henceforth labeled “horizontal” and “vertical analysis”). Analogously to SNARC experiments, the individual IES differences (dIES) for responses to right-/upward minus left-/downward target presentations were calculated for each target (i.e., mean RTs/PC for right-/upward presentations minus mean RTs/PC for left-/downward presentations for each target). Thus, negative dIES values indicate more efficient responses to rightward presentation in horizontal analyses and to upward presentation in the vertical analyses. The individual dIES regression slopes over targets were tested against zero (e.g., Lorch and Myers, 1990; Pfister et al., 2013). Regression slopes from the horizontal and vertical analyses were compared against each other with a paired *t*-test. Effect sizes for *t*-tests were computed as Cohen’s *d_z_* (cf. Lakens, 2013). All analyses were additionally conducted with RTs instead of IES for a more comprehensive assessment of the data.

For analyzing diagonal presentations, presentation positions on the target screen were segmented into four equal squares: left up, right up, left down, and right down (each including 3 × 3 mini-squares), excluding trials with targets on the respective central positions along the horizontal and vertical middle axis. Based on Hesse and Bremmer (2017), we calculated further dIES values: IES for right up-presentations minus IES for left down-presentations as well as IES for right down-presentations minus IES for left up-presentations per participant and target number. The resulting individual slopes over target numbers were again tested against zero.

## Results

**FIGURE 2:**
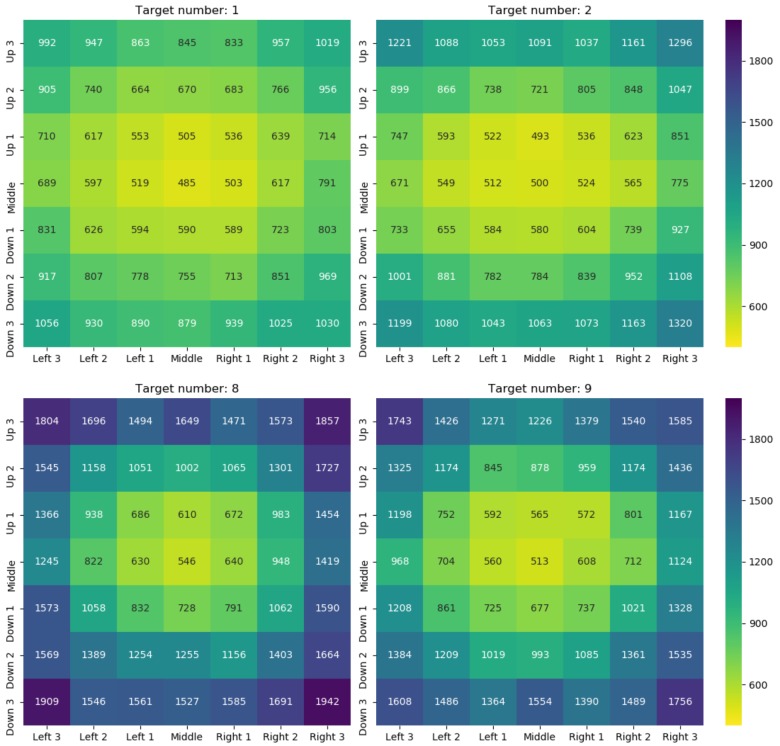
Mean IES (in ms) per target number (see headings) and spatial position within the 7 × 7 grid. Lower values/yellow color represent better performance and higher values/blue color represent worse performance (see color scale).

Preliminary analyses showed that in general IES (as well as RTs) increased with spatial distance of the target number from the center, all pairwise comparisons (Bonferroni-corrected) with *p* < 0.001, all *d_z_* > 1.61. Furthermore, large target numbers (i.e., 8, 9) had larger IES (as well as RTs) than small target numbers (i.e., 1, 2). On average, IES for large numbers were larger than IES for small numbers by 379 ms, *t*(29) = 15.43, *p* < 0.001, *d_z_* = 2.82. Figure 2 depicts all mean IES per target number and spatial position.

**FIGURE 3:**
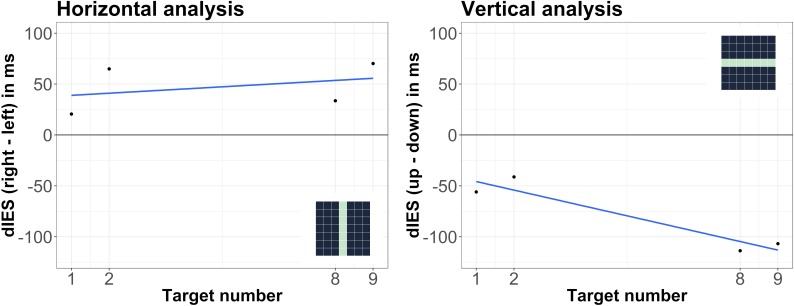
Differences between IES (dIES) when target numbers were presented at different locations on the screen. Left: left vs. right presentation, positive values indicate more efficient responses for left presentation; right: lower vs. upper presentation, negative values indicate more efficient responses for upper presentation. The spatial positions within the 7 × 7 grid that were compared against each other in the respective analysis are illustrated as dark areas in the depicted miniature 7 × 7 grids.

Horizontal and vertical analyses yielded a non-significant horizontal SNA and a significant vertical SNA: as visible in Figure 3, the slope in the horizontal analysis was not significantly different from zero, *t*(29) = 0.69, *p* = 0.497, but the slope in the vertical analysis was, *t*(29) = -2.28, *p* = 0.030, *d_z_* = 0.42. Moreover, the difference between the slopes was significant: the slopes from the vertical analysis were significantly larger (more negative) than the slopes from the horizontal analysis, *t*(29) = 2.10, *p* = 0.045, *d_z_* = 0.38. Analyses of RTs (instead of IES) yielded similar results: the slope in the horizontal analysis was not significantly different from zero, *t*(29) = 0.42, *p* = 0.677, but the slope in the vertical analyses was *t*(29) = -2.15, *p* = 0.040, *d_z_* = 0.39. However, the difference between the two slopes was not significant, *t*(29) = 1.88, *p* = 0.071. Overall, the results suggest that large numbers were more strongly associated with upper space than small numbers.

**FIGURE 4:**
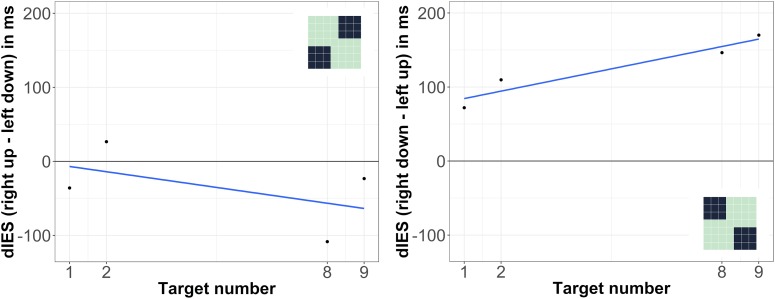
Differences between IES (dIES) when target numbers were presented at different locations on the screen. Left: left down vs. right up presentation, negative values indicate more efficient responses for right upper presentation; right: left up vs. right down presentation, positive values indicate more efficient responses for left upper presentation. The spatial positions within the 7 × 7 grid that were compared against each other in the respective analysis are illustrated as dark areas in the depicted miniature 7 × 7 grids.

In the diagonal analyses, the on average negative slope for the analysis regarding the left down-right up axis was statistically not significant, *t*(29) = -1.32, *p* = 0.196; also the on average positive slope for the analysis regarding the left up-right down axis was not significant, *t*(29) = 1.88, *p* = 0.070 (see Figure 4). Analyses of RTs (instead of IES) showed the same general – but non-significant – tendencies: *t*(29) = -1.27, *p* = 0.213 for the left down-right up axis; *t*(29) = 1.50, *p* = 0.144 for the left up-right down axis.

**FIGURE 5:**
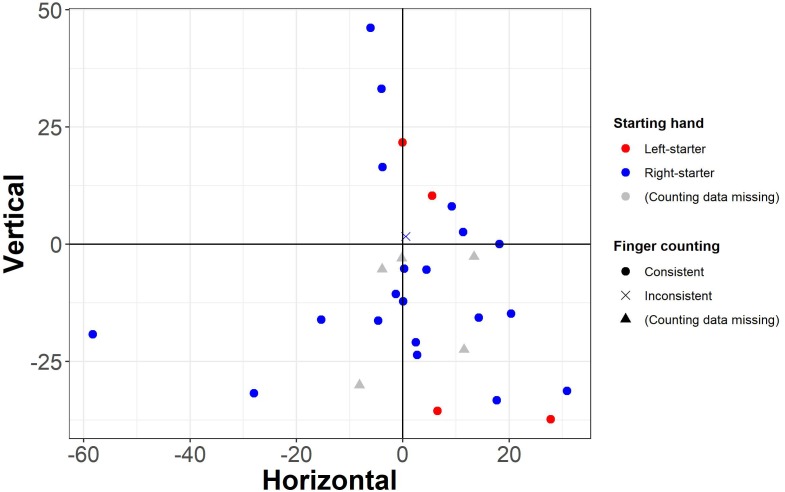
Individual slopes resulting from the horizontal and vertical analyses (see axis labels). Each data point represents one participant. Circles: consistent finger counters (from thumb to pinkie with both hands); cross: inconsistent finger counter (from index finger to pinkie with the starting hand). Red: left-starters; blue: right-starters; gray triangles: missing counting data.

To explore individual differences in mental number representations, we furthermore compared individual slopes of the horizontal and vertical analyses and found that the two did not significantly correlate, ρ = -0.097, *t*(28) = -0.52, *p* = 0.610. As visible in Figure 5, the present data suggest SNAs following a down-small to up-large association for most participants: 21 of the 30 participants (i.e., 70%) had negative slopes in the vertical analysis which indicate more efficient responses to up- than downward presentations for larger numbers relative to smaller numbers.

Regarding finger counting habits, four participants were left-starters in finger counting, 21 were right-starters, and for five this information is missing. Twenty-nine counted from thumb to pinkie with the starting and second hand for numbers 1–5 and 6–10, respectively (i.e., “consistent” counters). One participant counted from index finger to pinkie with the starting hand for numbers 1–4, used the full starting hand for number 5, and counted from thumb to pinkie of the second hand for numbers 6–10 (i.e., “inconsistent” counter). The effect of the starting hand and consistency of the fingers used in finger counting on horizontal and/or vertical SNAs was not statistically analyzed, because of the small sample size of left-starters (*n* = 4) and inconsistent counters (*n* = 1). Descriptively, the four left-starters did not share – and therefore did not point toward – a specific finger counting-dependent SNA pattern. Descriptive results are depicted in Figure 5.

## Discussion

With the present study, we investigated mental representations of numbers in two-dimensional space. Importantly, our study extended previous research by combining the two spatial dimensions within a single paradigm without imposing the response location and thereby priming spatial congruency relations.

First of all, we compared SNAs in horizontal and vertical space and found that large numbers were more strongly associated with upper space than small numbers, implying a bottom-small to top-large directionality. Horizontal associations, however, neither significantly followed a left-to-right nor right-to-left directionality. When we focused on diagonally arranged presentation positions, results were not as clear-cut. Reflecting the results from the vertical analysis, large numbers were tendentially more strongly associated with upper left and upper right space (in comparison to lower right and lower left space, respectively) than small numbers, but the slopes did not significantly differ from zero. Note, however, that each of the diagonal analyses included only a subset of the horizontal and vertical analyses. In an exploratory analysis, we furthermore compared individual SNAs for both horizontal and vertical associations and found no correlation between the two measures.

The dominance of the vertical association is in line with a recent study by Shaki and Fischer (2018) who argued that the horizontal SNARC effect “is an artifact of its measurement and number concepts are not inherently associated with horizontal space. The presence of horizontal SNAs (…) requires contextual priming” (p. 112). Regarding the vertical dimension, however, they provided “the first evidence for a purely conceptual SNA in this dimension” (p. 112). As in the present study, Shaki and Fischer’s (2018) experiment involved a Go/No-go task with only one central response button. Avoiding a spatial distribution of response buttons is essential to ensure that the spatial association under investigation is not created by the responses alone. The idea behind Shaki and Fischer’s (2018) study was that implicit associations between numbers and spatial concepts (arrows pointing toward different directions) influenced response efficiency. In the present experiment, the idea was that numbers influenced search behavior and/or spatial attention and thereby also response efficiency at different spatial locations. That is, the conceptualization of SNAs was not completely identical in that the spatial component in their case consisted of spatial concepts and in our case of spatial expectancies or attention shifts. Evidently, both kinds of SNAs exist with measurable effects in vertical space but not in horizontal space. The current results are also in line with the study by Blini et al. (2018) who found that mental arithmetic affected gaze positions. Here, SNAs refer to the relationship between gaze position and operation type. Vertical eye movements were more reliably affected (i.e., downward movements during subtractions and upward movements during additions) than horizontal eye movements. Taken together, evidence from a large variety of tasks is accumulating that vertical SNAs are more robust than horizontal SNAs.

On the other hand, the finding that the vertical association “trumped” the horizontal association seems to be in conflict with Holmes and Lourenco (2012; see also Hesse and Bremmer, 2017), where the horizontal association determined the SNARC slope more strongly than the vertical association when response buttons were arranged diagonally on the top left and bottom right. However, these divergent results are not surprising when taking into account Shaki and Fischer’s (2018) explanation that horizontal SNAs require contextual priming – which in this case consists in the presence of spatially arranged response buttons. Thus, horizontal SNAs can easily be primed and then may also be even stronger than vertical SNAs. Importantly, they fail to appear when their dimension is not primed while vertical SNAs persist. For a more general critique of the validity of diagonal SNAs, see Winter et al. (2015, p. 215).

Regarding the intra-individual comparison of horizontal and vertical SNAs, individual effects for horizontal and vertical associations did not seem to be related with one another: neither did participants have exclusive preferences for horizontal or vertical SNAs nor was there an “all-or-nothing” tendency with either both horizontal and vertical SNAs or none. Instead, about two-thirds of participants exhibited vertical SNAs while only about one-third (partly overlapping) exhibited horizontal SNAs in the expected directions. Interestingly, the percentage of participants exhibiting vertical SNAs corresponds to the percentage of participants exhibiting horizontal SNAs in SNARC experiments in which the horizontal dimension is primed by task demands (between 60–80%; cf. Cipora and Wood, 2017; Wood et al., 2006a,b, 2008). A next step will be to determine whether participants who are responsive to horizontal primes are the same who resort to the vertical dimension in 2D space when no dimension is primed. In fact, although the present study extends previous research by combining the horizontal and vertical axis, it is still limited in its validity regarding individual conceptual SNAs. First of all, there is still one spatial dimension missing before comprehensive conclusions can be drawn regarding associations between numbers and all of space. Moreover, it only regards a limited range of numbers. As shown by Galton (1880), spatial associations of larger numbers can deviate substantially from those of single-digit numbers as used in the present experiment. Furthermore, mental space might not be as linearly arranged as the space employed by any kind of experiment. The best that can be done experimentally is to approach conceptual SNAs as closely as possible. As also shown by the present experiment, the exact orientation of SNAs seems to be a very idiosyncratic property with a more frequent occurrence of a preference for large numbers spatially above small numbers.

Furthermore, an as yet unmentioned finding of the present experiment was that responses were overall more efficient for target presentations in left and in upper space. A leftward bias might partly be explained by pseudoneglect, that is, an attentional bias toward left space in healthy persons (e.g., Jewell and McCourt, 2000). However, the fact that responses were also more efficient in upper space might suggest that visual search was affected by reading direction, which would be expected to begin at the upper left in 2D space. However, we were mainly interested in the relative efficiency of small and large numbers in 2D space, which is why we will not go into detail regarding general search behavior in our task.

The mechanism behind the reported SNA effect, that is, relatively faster RTs for large numbers in upper space, presumably involves an attentional shift and faster and/or preferred saccades toward the associated spatial position. Evidence comes from studies investigating horizontal SNAs for visually presented numbers. In a study by Fischer et al. (2003), visual attention was shifted toward the left or right side by mere visual perception of Arabic digits. While this seminal finding is now under scrutiny (cf. Fischer and Knops, 2014), attentional consequences of number processing have now been extensively documented also in mental arithmetic (review in Fischer and Shaki, 2018). In addition, Fischer et al. (2004) investigated gaze durations after visual number presentations. Participants had to perform saccades to the left or right side of a centrally presented Arabic digit depending on the digit’s parity. In responses to small numbers, leftward saccades were initiated faster than rightward saccades and vice versa for large numbers. Future studies employing spatially distributed target numbers in two-dimensional space could integrate eye tracking to further explore the impact of number magnitude on search behavior.

## Conclusion

The present study fills a gap as yet untouched by previous research: by arranging stimuli within a two-dimensional grid and thereby avoiding to prime any single axis, we extended studies on horizontal, vertical, and diagonal SNAs. Our main finding was that SNAs were predominantly determined by the vertical axis – with large numbers being more strongly associated with upper space than small numbers – while there was no specific preference for small vs. large numbers on the left vs. right side. Moreover, individual effects differed and we reported the relation of horizontal and vertical associations on an individual basis. Taken together, numbers seem to be conceptually associated with vertical but not horizontal space when number magnitude is task-irrelevant and neither spatial dimension is primed by task demands.

## Author Contributions

ES, JL, and KW contributed to conception and design of the study. ES programmed the experiments, performed the statistical analysis, and wrote the first draft of the manuscript. All authors contributed to manuscript revision, and read and approved the submitted version.

## Conflict of Interest Statement

The authors declare that the research was conducted in the absence of any commercial or financial relationships that could be construed as a potential conflict of interest.
